# Induction of γ‐aminobutyric acid plays a positive role to *Arabidopsis* resistance against *Pseudomonas syringae*

**DOI:** 10.1111/jipb.12974

**Published:** 2020-06-26

**Authors:** Xiangxiong Deng, Xuwen Xu, Yu Liu, Yan Zhang, Liuyi Yang, Shuqun Zhang, Juan Xu

**Affiliations:** ^1^ State Key Laboratory of Plant Physiology and Biochemistry, College of Life Sciences Zhejiang University Hangzhou 310058 China; ^2^ Division of Biochemistry University of Missouri Columbia Missouri 65211 USA

## Abstract

Gamma‐aminobutyric acid (GABA) is an important metabolite which functions in plant growth, development, and stress responses. However, its role in plant defense and how it is regulated are largely unknown. Here, we report a detailed analysis of GABA induction during the resistance response to *Pseudomonas syringae* in *Arabidopsis thaliana*. While searching for the mechanism underlying the pathogen‐responsive mitogen‐activated protein kinase (MPK)3/MPK6 signaling cascade in plant immunity, we found that activation of MPK3/MPK6 greatly induced GABA biosynthesis, which is dependent on the glutamate decarboxylase genes *GAD1* and *GAD4*. Inoculation with *Pseudomonas syringae* pv *tomato* DC3000 (*Pst*) and *Pst‐avrRpt2* expressing the *avrRpt2* effector gene induced *GAD1* and *GAD4* gene expression and increased the levels of GABA. Genetic evidence revealed that *GAD1, GAD2*, and *GAD4* play important roles in both GABA biosynthesis and plant resistance in response to *Pst‐avrRpt2* infection. The *gad1/2/4* triple and *gad1/2/4/5* quadruple mutants, in which the GABA levels were extremely low, were more susceptible to both *Pst* and *Pst‐avrRpt2*. Functional loss of MPK3/MPK6, or their upstream MKK4/MKK5, or their downstream substrate WRKY33 suppressed the induction of *GAD1* and *GAD4* expression after *Pst‐avrRpt2* treatment. Our findings shed light on both the regulation and role of GABA in the plant immunity to a bacterial pathogen.

## Graphical abstract

GABA (γ‐aminobutyric acid), a ubiquitous four‐carbon amino acid, is rapidly induced in plants during their interaction with pathogens. Here, we demonstrate that induction of GABA bolsters the *Arabidopsis* resistance response to a bacterial pathogen. The GABA balance is important to both plant growth and disease resistance.

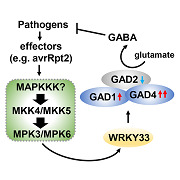

## INTRODUCTION

Gamma‐aminobutyric acid (GABA) is a ubiquitous, four‐carbon, non‐proteinogenic amino acid which widely exists in bacteria, plants, and animals. GABA functions as an important signaling molecule and a trophic metabolite. Gamma‐aminobutyric acid is synthesized from glutamate and degraded to succinate through a short pathway called the GABA shunt. The evolutionarily conserved glutamate decarboxylase (GAD) converts glutamate to GABA. Gamma‐aminobutyric acid then undergoes a two‐step reaction, catalyzed by GABA transaminase (GABA‐T) and succinic semialdehyde dehydrogenase (SSADH), respectively, to form succinate, which can re‐enter the tricarboxylic acid cycle (TCA cycle) (Bown and Shelp 1997; Shelp et al. 1999; Bouche et al. 2003; Bouche and Fromm 2004; Fait et al. 2008). In plants, GAD‐mediated GABA generation is the major source of GABA (Fait et al. 2008; Shelp et al. 2012). Either activation of GAD or inhibition of GABA‐T or SSADH can lead to the accumulation of cellular GABA.

In plants, GABA primarily serves as an intermediate metabolite in primary C/N metabolism through the TCA cycle (Bown and Shelp 1997; Shelp et al. 1999; Bouche and Fromm 2004; Fait et al. 2008; Michaeli and Fromm 2015). Emerging evidence has indicated that GABA also serves as a signaling molecule in plants, as it does in animals. It has long been reported that there are potential GABA receptors present on the plant protoplast membrane (Yu et al. 2006). A candidate GABA receptor, the plant‐specific aluminium‐activated malate transporter (ALMT), was identified (Ramesh et al. 2015). This report showed that GABA signaling modulates plant growth under both stressed and nonstressed conditions by directly regulating the activity of ALMT.

Gamma‐aminobutyric acid has also been reported to play roles in various physiological processes in plants. During plant growth and development, GABA is essential during fruit and seed development, root growth, senescence, and hormone regulation (Akihiro et al. 2008; Fait et al. 2011; Renault et al. 2013; Sun et al. 2013). Gamma‐aminobutyric acid gradients were reported to be required for pollen tube growth and guidance (Palanivelu et al. 2003; Renault et al. 2011; Yu et al. 2014). Plants under either biotic stresses, such as animal/insect herbivory and microbial infection, or abiotic stresses, such as hypoxia, salt, cold, and drought, were all found to show increased cellular GABA levels (Bown and Shelp 1997; Shelp et al. 1999; Bown et al. 2006; Li et al. 2019). Increased GABA shunt activity is associated with increased resistance to *Agrobacterium* in tobacco or to *Botrytis cinerea* in tomato (Chevrot et al. 2006; Seifi et al. 2013). In addition, the *Pseudomonas syringae* pv *tomato* (*Pst*) *gabT* triple mutant strain, which cannot degrade GABA, was found to be less virulent. Virulence of the *Pst gabT* strain was further reduced when inoculated in the *Arabidopsis pop2/gaba‐t* mutant, which accumulates a higher level of GABA (Park et al. 2010). These results indicated that GABA could play a positive role in plant immunity. However, the function of GABA in plant defense and the underlying mechanism regulating GABA biosynthesis remain largely unknown.

The highly conserved mitogen‐activated protein kinase (MAPK or MPK) signaling pathways play pivotal roles in plant growth, development and defense (Pitzschke et al. 2009; Rodriguez et al. 2010; Tena et al. 2011; Meng and Zhang 2013; Zhang et al. 2018). MPKs are key signaling modules downstream of the cellular receptors and sensors that perceive endogenous/exogenous stimuli, including pathogen‐derived molecular patterns and effectors. In *Arabidopsis*, MPK3 and MPK6, two functionally redundant MAPKs that act downstream of two redundant MAPKKs (MKK4 and MKK5), regulate defense responses including stomatal immunity, ethylene biosynthesis, defense chemical accumulation, hypersensitive response‐initiated cell death, and defense gene activation (Meng and Zhang 2013; Doczi and Bogre 2018; Zhang et al. 2018). Activation of MPK3/MPK6 is one of the earliest signaling events after a plant senses pathogen invasion. MPK3/MPK6 are transiently activated during pattern‐triggered immunity (PTI), but are activated in a more robust and long‐lasting way during effector‐triggered immunity (ETI) (Tsuda et al. 2013; Guan et al. 2015). The different kinetics (magnitude and duration) of MAPK activation could lead to differential outcomes during PTI and ETI. The outcome of MAPK cascade activation also depends on the availability of downstream MAPK substrates, including transcription factors. One such downstream transcription factor is WRKY33, which is involved in phytoalexin biosynthesis and ethylene induction during *Arabidopsis* immunity (Mao et al. 2011; Li et al. 2012).

In this study, we found that GABA is greatly induced during ETI or following activation of the MPK3/MPK6 signaling cascade, which is associated with high *GAD1* and *GAD4* gene expression. We generated GABA loss‐of‐function mutants and gain‐of‐function transgenic plants and demonstrated that GABA balance is critical in both plant resistance and growth. Genetic and disease analyses revealed that the induction of GABA, which is dependent on *GAD1*, *GAD2*, and *GAD4*, plays a positive role in both PTI and ETI. Over‐accumulation of GABA in the transgenic plants with elevated GAD activity greatly suppressed plant growth. In response to *Pst‐avrRpt2* inoculation, expression of *GAD1* and *GAD4* is regulated by the MKK4/MKK5‐MPK3/MPK6 cascades, specifically through the downstream WRKY33 transcription factor.

## RESULTS

### Activation of MPK3/MPK6 highly induces *GAD* expression

**Figure 1:**
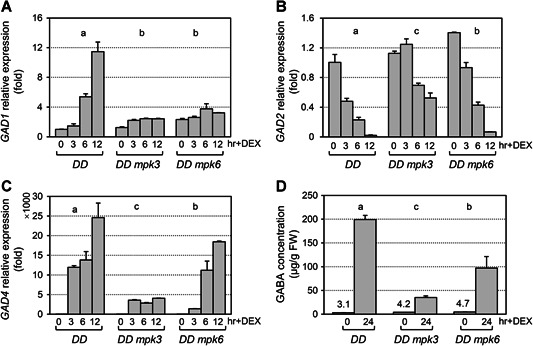
**Activation of mitogen‐activated protein kinase (MPK)3/MPK6 induces the expression of *glutamate decarboxylase (GAD)1* and *GAD4* genes and leads to high level of gamma‐aminobutyric acid (GABA) accumulation** (**A–C**) Fourteen‐d‐old seedlings grown in gas chromatography vials supplied with swimming medium were collected at the indicated times after addition of 5 μmol/L dexamethasone (DEX). After reverse transcription, levels of *GAD1* (**A**), *GAD2* (**B**), and *GAD4* (**C**) transcripts were determined by real‐time quantitative polymerase chain reaction (PCR). Relative expression was analyzed by two‐way analysis of variance (ANOVA) (genotype × time point). Different lowercase letters above the groups indicate statistically different groups (*P* < 0.0001). Error bars indicate *SD* (*n* = 3). (**D**) Fourteen‐d‐old, soil‐grown seedlings were sprayed with 30 μmol/L DEX. Leaves were collected at the indicated times after application of DEX. Gamma‐aminobutyric acid accumulation was determined using a Hitachi Automatic Amino Acid Analyzer, L‐8900. Gamma‐aminobutyric acid concentration was analyzed by two‐way ANOVA (genotype × time point). Different lowercase letters above the groups indicate statistically different groups (*P* < 0.001). The numbers above each bar at time zero indicate the low initial GABA level (μg/g FW). Error bars indicate *SD* (*n* = 3), FW, fresh weight.

*Arabidopsis* MPK3 and MPK6 are rapidly activated after pathogen invasion is detected and function redundantly to activate several defense responses, including defense gene expression, phytoalexin biosynthesis, ethylene induction, and stomatal immunity (Meng and Zhang 2013; Zhang et al. 2018). To identify unknown components downstream of the MPK3/MPK6 cascade during plant immunity, we remined the expression profiling data in *GVG*‐*NtMEK2* ^*DD*^ transgenic plants (abbreviated as *DD*), in which MPK3/MPK6 can be continuously activated by dexamethasone (DEX) treatment (Su et al. 2018). With DEX‐activation of MPK3/MPK6, *GAD4* was one of the most highly induced genes. There are five members in the *GAD* gene family in *Arabidopsis thaliana* (Shelp et al. 1999). The expression of all five *GAD* genes in *DD* seedlings after DEX treatment was tracked using quantitative reverse transcription polymerase chain reaction (RT‐qPCR) (Figure 1). The expression levels of both *GAD1* and *GAD4* were greatly upregulated, while expression of *GAD2* was downregulated (Figure 1A–C). Under normal conditions, *GAD2* is the most abundant GAD transcript in *Arabidopsis* leaves (Miyashita and Good 2008). Within 12 h after activation of MPK3/MPK6 in DEX‐treated *DD* seedlings, *GAD4* increased by about 23 000‐fold, whereas *GAD1* was induced about 15‐fold. In *DD* plants in either the *mpk3* or *mpk6* mutant background, the induction of *GAD1/4* was reduced, as was the suppression of *GAD2* (Figure 1A–C). This indicated that MPK3/MPK6 signaling is necessary for regulated expression of these *GAD* genes. *GAD3* and *GAD5* transcripts were not detected in *DD* seedlings.

The expression pattern of these five *GAD* promoters were analyzed in seedling tissues utilizing the β‐glucuronidase (GUS) reporter. Observation of more than 40 transgenic lines of the T1 generation for each promoter‐GUS fusion indicated that *GAD1*, *GAD2*, and *GAD4* were mainly expressed in leaves and roots at the vegetative stage (Figure S1). Although *GAD3* and *GAD5* transcripts were not detected in whole seedlings using RT‐qPCR, *GAD5* expression was visible in the pollen, and *GAD3* in the anthers and embryos utilizing the GUS reporter. The differential expression pattern and differential induction kinetics of the *GAD* genes after activation of MPK3/MPK6 indicated they may play different roles in the spatio‐temporal biosynthesis of GABA in plants.

### **Activation of MPK3/MPK6 leads to very high levels of GABA, which is genetically dependent on** *GAD1* **and** *GAD4*

To examine if MPK3/MPK6 signaling plays a role in GABA induction, the GABA levels were determined in *DD* plants after DEX treatment (Figure 1D). In the *DD* line, GABA was induced ~70‐fold 24 h after DEX treatment. This induction was greatly compromised in either the *mpk3* or *mpk6* mutant background, especially in *mpk3*. These results indicated that activation of MPK3/MPK6 greatly promoted GABA accumulation and that MPK3 and MPK6 function redundantly in regulation of GABA biosynthesis.

**Figure 2:**
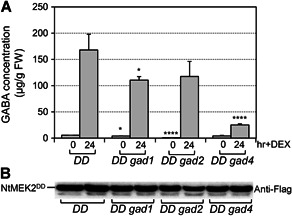
**Gamma‐aminobutyric acid (GABA) induction after activation of mitogen‐activated protein kinase (MPK)3/MPK6 in *glutamate decarboxylase (gad)1*, *gad2* and *gad4* mutants** (**A**) Fourteen‐d‐old *DD*, *DD gad1*, *DD gad2*, and *DD gad4* seedlings were sprayed with 30 μmol/L dexamethasone (DEX). Samples were collected 24 h later for GABA analysis. One‐way analysis of variance (ANOVA) was performed to compare different genotypes with *DD* at the same time point (*P < 0.05; ****P < 0.0001). Error bars indicate *SD* (*n* = 3), FW, fresh weight. (**B**) Levels of the Flag‐tagged NtMEK2^DD^ protein in the different lines were detected by immunoblot analysis using an anti‐Flag antibody.

In order to genetically determine which *GAD* gene is responsible for MPK3/MPK6‐mediated GABA induction, *gad1*, *gad2*, and *gad4* knockout mutants that were obtained from the *Arabidopsis* Biological Resource Center (ABRC) (Figure S2) were independently crossed into the *DD* background and carried forward to create homozygous *DD gad1*, *DD gad2*, and *DD gad4* plants. GABA concentrations were examined in these *DD gad* mutants (Figure 2). Loss of function of *gad1* or *gad4* led to a 35% or 85% loss of GABA induction, respectively, after MPK3/MPK6 activation. Mutation of *gad2* had no significant effect on GABA induction in *DD* plants, but did reduce the baseline GABA level (from 5.5 μg/g in *DD* to 0.8 μg/g in *DD gad2* without DEX treatment). Taken together, these results indicated that MPK3/MPK6 signaling regulates GABA biosynthesis mainly through *GAD1* and *GAD4*.

### **Pathogen inoculation induces a high level of GABA accumulation and** *GAD1/GAD4* **induction, especially during avrRpt2‐triggered immunity**

**Figure 3:**
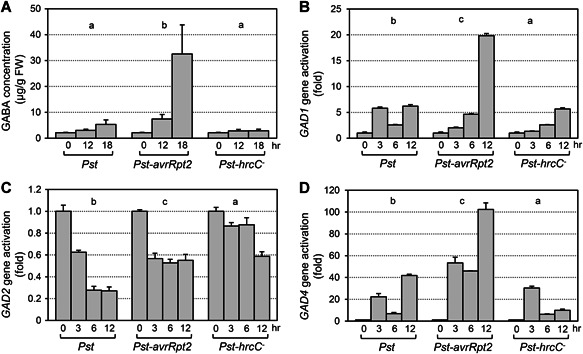
**Gamma‐aminobutyric acid (GABA) level and *glutamate decarboxylase (GAD)* gene expression after *Pst*, *Pst‐hrcC*^*‐*^, or *Pst‐avrRpt2* inoculation in *Arabidopsis*** (**A**) Fourteen‐d‐old seedlings of Col‐0 were sprayed with *Pst*, *Pst‐hrcC* ^*‐*^, or *Pst‐AvrRpt2* (final OD_600_ = 0.4). The shoots of the inoculated seedlings were collected at the indicated times. Gamma‐aminobutyric acid levels were determined using a Hitachi Automatic Amino Acid Analyzer. FW, fresh weight. (**B–D**) Fourteen‐d‐old seedlings were collected at the indicated times after inoculation with *Pst*, *Pst‐hrcC* ^*‐*^, or *Pst‐AvrRpt2*. After reverse transcription, the levels of *GAD1* (**B**), *GAD2* (**C**), and *GAD4* (**D**) transcripts were determined by real‐time quantitative polymerase chain reaction (PCR). Gamma‐aminobutyric acid concentration and *GAD* relative expression were analyzed by two‐way analysis of variance (treatment × time point). Different lowercase letters above the groups indicate statistically different groups (*P* < 0.0001). Error bars indicate *SD* (*n* = 3).

The MPK3/MPK6 signaling cascade is highly responsive to infection by pathogens, including *Pseudomonas syringae* pv *tomato* DC3000 (*Pst*), a model bacteria widely used for studying plant disease resistance (Tsuda et al. 2013; Guan et al. 2015). To better understand the role of MPK3/MPK6 in regulating GABA biosynthesis during plant immunity against *Pst* infection, the levels of GABA and the involved *GAD* genes were measured in wild‐type Col‐0 after *Pst*, *Pst‐avrRpt2*, and *Pst‐hrcC* ^*‐*^ inoculation (Figure 3A). Infection with *Pst* led to about a 2.5‐fold higher accumulation of GABA at 18 h. There was no obvious change in GABA levels over time in response to *Pst‐hrcC* ^*‐*^, a *Pst* strain that cannot deliver effectors into plant cells due to a deletion affecting the Type III Secretion System. On the other hand, inoculation with *Pst‐avrRpt2*, a strain expressing the *avrRpt2* effector gene that can activate MPK3/MPK6 in a long‐lasting way (Guan et al. 2015), induced GABA by 20‐fold at 18 h after infection (Figure 3A). This high level of GABA induction in response to *Pst‐avrRpt2* infection is likely a result of avrRpt2 ETI.

*Glutamate decarboxylase* expression was monitored by RT‐qPCR in wild‐type seedlings over time after inoculation with *Pst*, *Pst‐avrRpt2*, and *Pst‐hrcC* ^*‐*^ (Figure 3B–D). Both *GAD1* and *GAD4* were highly induced within 12 h by *Pst‐avrRpt2*, by about 20‐fold and 100‐fold, respectively. *Pst* and *Pst‐hrcC* ^*‐*^ also induced *GAD1* and *GAD4* gene expression, but to a relatively lower level. Similar to the pattern in *DD* plants, expression of *GAD2* was downregulated after *Pst*, *Pst‐avrRpt2*, or *Pst‐hrcC* ^*‐*^ infection. The differential expression pattern of these *GAD* genes after pathogen invasion suggested that they might play different roles during plant defense responses.

### **During** *Pst‐avrRpt2* **infection, expression of** *GAD1/4* **is regulated by the MKK4/MKK5‐MPK3/MPK6 cascade and the downstream transcription factor WRKY33**

**Figure 4:**
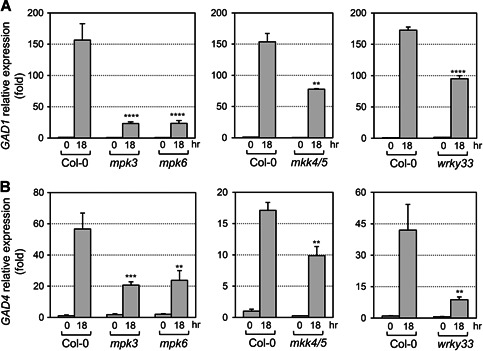
***Pst‐avrRpt2*‐induced transcript accumulation of *glutamate decarboxylase (GAD)1* and *GAD4* were compromised in *mpk3*, *mpk6*, *mkk4 mkk5*, and *wrky33* mutants** (**A, B**) Fourteen‐d‐old seedlings were collected at indicated times after spraying with *Pst‐avrRpt2* (OD_600_ = 0.4). Gene expression was quantified by quantitative reverse transcription polymerase chain reaction (RT‐qPCR). One‐way analysis of variance (ANOVA) was performed when three genotypes were compared, and Student's *t*‐test was performed when two genotypes were compared. Asterisks above the columns indicate statistical difference compared to Col‐0 (**P < 0.01; ***P < 0.001; ****P < 0.0001). Error bars indicate *SD* (*n* = 3).

To determine the role of MPK3 and MPK6 in *Pst‐avrRpt2*‐induced GABA production, *GAD* gene expression was measured in *mpk3* and *mpk6* single mutants after *Pst‐avrRpt2* inoculation (Figure 4). Expression of *GAD1* and *GAD4* was significantly reduced in the *mpk3* and *mpk6* mutants compared to the levels seen in wild type, demonstrating that *GAD1* and *GAD4* upregulation is downstream of MPK3/MPK6 signaling during pathogen infection. Expression of *GAD2* remained at the same level or even increased in the *mpk3* and *mpk6* single mutants after *Pst‐avrRpt2* inoculation (Figure S3), implying that *GAD2* might be negatively regulated by MPK3/MPK6 during plant immunity.

Our previous study illustrated that MKK4 and MKK5 function upstream of MPK3/MPK6 to regulate plant defense responses (Su et al. 2017). In order to figure out whether MKK4 and MKK5 function as MAPKKs upstream of MPK3/MPK6 during regulation of *GAD* gene expression, *GAD* gene expression was analyzed in the *mkk4 mkk5* double mutant after *Pst‐avrRpt2* inoculation (Figure 4). We found the induction of *GAD1* and *GAD4* gene expression was significantly compromised in *mkk4 mkk5* after *Pst‐avrRpt2* inoculation, similar to that in the *mpk3* or *mpk6* mutant. This result indicated that MKK4 and MKK5 are part of the upstream signaling that regulates expression of *GAD1* and *GAD4*.

WRKY33, a substrate of MPK3/MPK6, was reported to regulate downstream genes to produce ethylene and antimicrobial chemicals in plant defense against bacterial or fungal pathogens (Mao et al. 2011; Li et al. 2012; Han et al. 2019). *Glutamate decarboxylase1/4* gene expression was investigated in the *wrky33* mutant before and after *Pst‐avrRpt2* inoculation. The *wrky33* mutation compromised *GAD1* and *GAD4* gene induction, implying that WRKY33 is working downstream of MKK4/MKK5‐MPK3/MPK6 signaling to regulate *GAD1/4* gene expression (Figure 4). This result is consistent with the recent chromatin immunoprecipitation sequencing result, which showed that the *GAD1* gene is a direct target of WRKY33 in response to flg22 treatment (Birkenbihl et al. 2017). In addition, similar to that in the *mpk3* and *mpk6* mutants, suppression of *GAD2* expression by *Pst‐avrRpt2* was blocked in both the *mkk4 mkk5* and *wrky33* mutants (Figure S3), suggesting again the negative regulation of *GAD2* expression by MKK4/MKK5‐MPK3/MPK6‐WRKY33 pathway.

### *Glutamate decarboxylase1/2/4* **are responsible for GABA accumulation in** *Arabidopsis* **shoots after** *Pst‐avrRpt2* **treatment**

**Figure 5:**
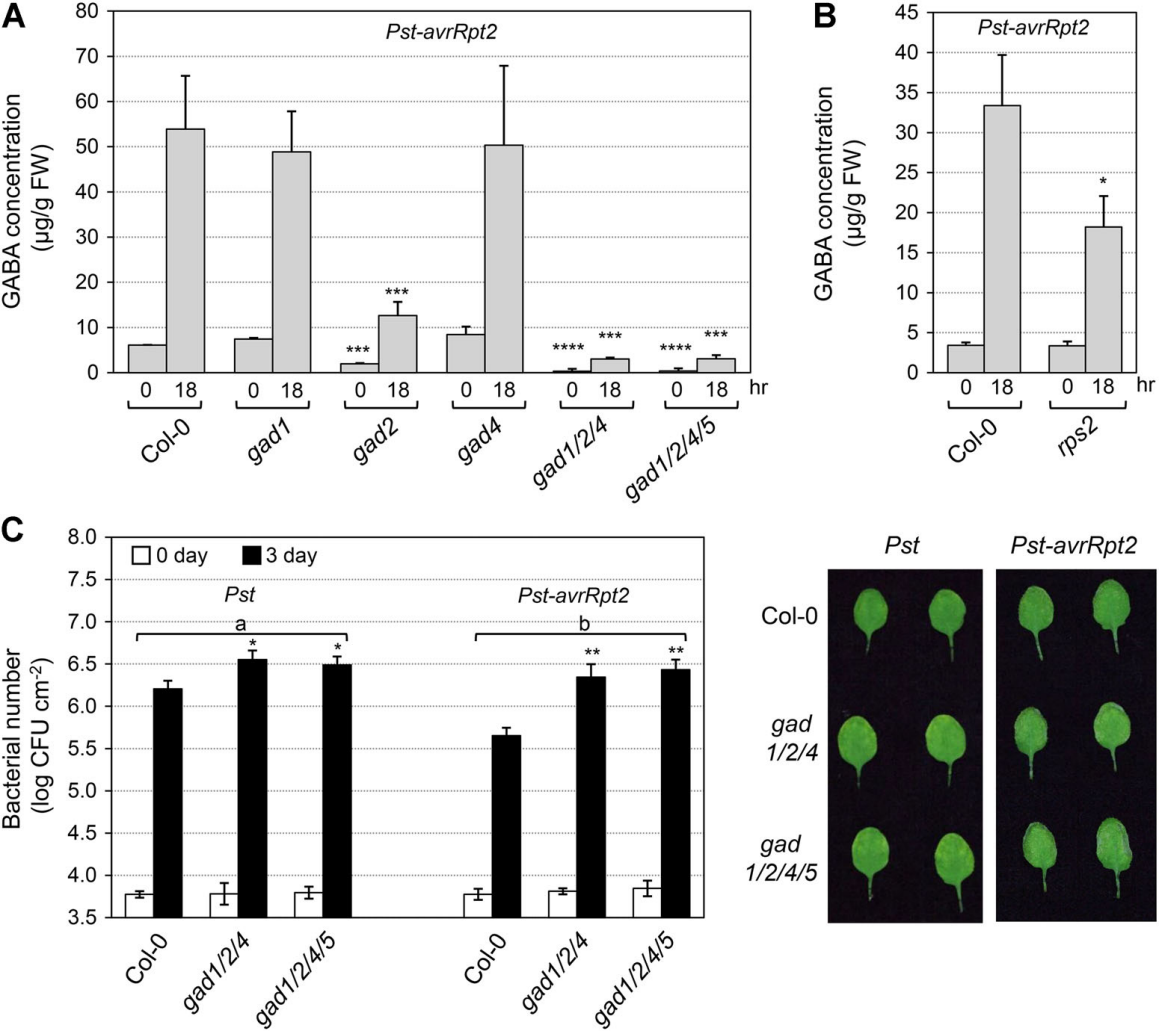
***Glutamate decarboxylase (GAD)1*, *GAD2*, and *GAD4* genes are important for both γ‐aminobutyric acid (GABA) induction and resistance to *Pst* and *Pst‐avrRpt2*** (**A, B)** Fourteen‐d‐old seedlings of Col‐0, various *gad* mutants, and *rps2* were sprayed with *Pst‐AvrRpt2* (final OD_600_ = 0.4). The shoots of the inoculated seedlings were collected at the indicated times. Gamma‐aminobutyric acid levels were determined using a Hitachi Automatic Amino Acid Analyzer. One‐way analysis of variance (ANOVA) (**A)** and Student's *t*‐test (**B)** were performed to compare different genotypes with the wild type at each time point. Asterisks above the columns indicate statistical difference (*P < 0.05; ***P < 0.001; ****P < 0.0001). Error bars indicate *SD* (*n* = 3). FW, fresh weight. (**C**) The leaves of 3‐week‐old plants were infiltrated with *Pst* and Pst‐avrRpt2 (OD_600_ = 0.001). Bacteria levels were quantified 0 and 3 days post‐inoculation (dpi). Differences in bacterial growth between *Pst* and *Pst‐avrRpt2* were analyzed by two‐way ANOVA, with different lowercase letters above the groups indicating statistically significant differences (*P* < 0.001). One‐way ANOVA was also performed to compare mutants with the wild type at the same time point (*P < 0.05; **P < 0.01). Error bars indicate *SD* (*n* = 3). CFU, colony‐forming units.

To further understand the role of each *GAD* gene in GABA production during plant immunity, GABA concentrations were examined in various *gad* mutants after *Pst‐avrRpt2* inoculation. In addition to the single knockout mutants of *gad1*, *gad2*, *gad4*, and *gad5* (Figure S2), crosses between the *gad* mutants were generated for analyses. A single loss of function in *gad1* or *gad4* had no effect on GABA biosynthesis under normal and pathogen‐inoculated conditions, while the GABA level was significantly decreased in the *gad2* mutant under both conditions (Figure 5A). Although *GAD2* was not induced with *Pst‐avrRpt2* inoculation (Figure 3C), *GAD2* does seem to have an important role in sustaining both basal and pathogen‐induced GABA levels. This is consistent with data showing that *GAD2* is the most abundant transcript among all *GAD* members in *Arabidopsis* leaves (Miyashita and Good 2008). The GABA level was further reduced to a very low level in the *gad1/2/4* triple mutant. In the *gad1/2/4/5* quadruple mutant, the GABA level was similar to that in the *gad1/2/4* triple mutant. These results indicated that *GAD1* and *GAD4* also make important contributions in GABA biosynthesis and work together with *GAD2* to regulate GABA levels in *Arabidopsis* in response to *Pst‐avrRpt2* infection.

### **Reduced GABA biosynthesis compromises plant resistance to** *Pst* **and** *Pst‐avrRpt2*

During the plant response to inoculation with either *Pst* or *Pst‐avrRpt2*, GABA levels were elevated (Figure 3A), suggesting a potential role of GABA in plant immunity. During the response to *Pst‐avrRpt2*, there was a dramatic induction of GABA accumulation. To further reveal if this sharp induction of GABA is an ETI‐related response, we measured *Pst‐avrRpt2*‐induced GABA accumulation in the *rps2* mutant seedlings, which lacks the R protein necessary to sense the avrRpt2 effector. In *rps2*, the GABA induction was significantly compromised as compared to the wild type (Figure 5B). However, the GABA induction was not completely blocked in the *rps2* mutant, which could indicate that some of the GABA biosynthesis is induced through PTI. This result suggested that the higher level of GABA induction is the result of an effector‐triggered response, and induction of GABA may play a role in both PTI and ETI.

To further understand how increased GABA levels function during the plant immunity response, we tested pathogen resistance in the *gad1/2/4* triple and *gad1/2/4/5* quadruple mutants, which exhibit no growth defect but accumulate much lower levels of GABA than the wild‐type plants. The *gad1/2/4* triple and *gad1/2/4/5* quadruple mutants showed enhanced susceptibility to both *Pst* and *Pst‐avrRpt2* infection (Figure 5C), but with differentially compromised resistance levels. The increase in *Pst* growth in the *gad1/2/4* and *gad1/2/4/5* mutants compared to Col‐0 was small (~0.4 log), but significant (*P* < 0.05). In contrast, the growth of *Pst‐avrRpt2* was ~0.8 log higher in the *gad1/2/4* and *gad1/2/4/5* mutants than in Col‐0. These results indicated that induction of GABA plays a positive role in both plant PTI and ETI.

In addition to GABA levels, the levels of several free amino acids that are abundant in *Arabidopsis* leaves were also analyzed (Hildebrandt et al. 2015), including aspartic acid (Asp), glutamate (Glu), glutamine (Gln), alanine (Ala), serine (Ser), and threonine (Thr). Eighteen hours after *Pst‐avrRpt2* infection, the levels of five of these amino acids (with the exception being Asp) increased in wild‐type plants (Figure S4). When GABA biosynthesis was blocked in the *gad1/2/4* triple or *gad1/2/4/5* quadruple mutant, the induction of Glu, Gln, Ser, and Thr was slightly reduced as compared to that in the wild type. However, the level of Ala, the main by‐product when GABA is catabolized to succinate and available to enter the TCA cycle (Figure S5), was only ~25%–35% of that in wild type in both mutants after *Pst‐avrRpt2* infection. Reduced Ala levels in the mutants could be a result of the reduced GABA catabolism.

### Over‐accumulation of GABA in plants leads to both physical and disease resistance phenotypes

**Figure 6:**
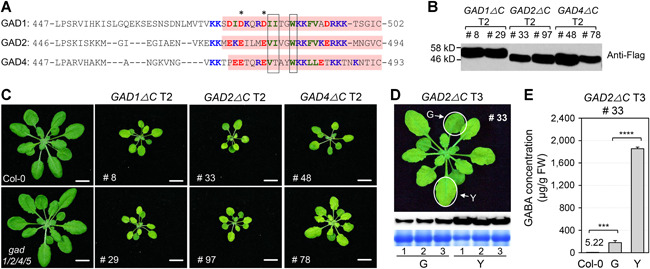
**Over‐accumulation of γ‐aminobutyric acid (GABA) in plants leads to a dwarf and yellowish phenotype** (**A**) Amino acid sequence alignment of the CaM‐binding domains of glutamate decarboxylase (GAD) proteins in *Arabidopsis*. Conserved acidic (red), basic (blue) and hydrophobic (green) residues are colored, key anchors are boxed, and potential pseudo‐substrate glutamate/aspartate residues are indicated with asterisks. Deleted residues in transgenic plants are shown with pink backgrounds. (**B**) Expression of GAD1ΔC, GAD2ΔC, and GAD4ΔC in T2 transgenic plants was detected by immunoblot analysis using anti‐Flag antibody. (**C**) Expression of GAD1ΔC, GAD2ΔC, and GAD4ΔC in T2 transgenic plants leads to dwarf and yellowish morphological phenotypes. Images were taken at 3 weeks. Bar = 1 cm. (**D**) Expression of GAD2ΔC in G (green) and Y (yellow) leaves of transgenic line #33 in the T3 generation was detected by immunoblot analysis using anti‐Flag antibody. The Coomassie‐stained gel is shown below as a loading control. The three pairs of G and Y leaves were from three different plants, respectively. Bar = 1 cm. (**E**) Gamma‐aminobutyric acid concentration in G (green) and Y (yellow) leaves of T3 transgenic line #33. Error bars indicate *SD* (*n* = 3). Gamma‐aminobutyric acid levels were analyzed by one‐way analysis of variance (ANOVA). Asterisks above the columns indicate statistical difference (***P < 0.001; ****P < 0.0001). Error bars indicate *SD* (*n* = 3).

Since low GABA levels in plants result in more susceptibility to *Pst‐avrRpt2*, the impact of over‐accumulation of GABA was investigated. The GAD enzyme can be activated by Ca^2+^/CaM binding to its C‐terminal auto‐inhibitory domain. The deletion of this ~50 amino acid C‐terminal domain can result in constitutively active GAD enzymes (Baum et al. 1996; Yap et al. 2003). We generated transgenic lines that overexpress truncated *AtGAD1/2/4* versions under the control of the CaMV 35S promoter (Figure 6A). These transgenic lines were named *GAD1ΔC*, *GAD2ΔC*, and *GAD4ΔC*, respectively. A Flag tag was attached to the N‐terminal of these truncated GADs for detecting protein levels (Figure 6B). At least two independent T2 lines with a single insertion for the respective transgene were selected for further analyses. All of these transgenic seedlings had a dwarf and yellowish phenotype in the T2 generation (Figure 6C). However, in the T3 generation, the *GAD1ΔC* and *GAD4ΔC* transgenic seedlings showed wild‐type morphology, which was associated with gene silencing. In contrast, the *GAD2ΔC* T3 seedlings still showed transgene expression and exhibited a yellow‐leafed phenotype. As a result, we focused on *GAD2ΔC* transgenic lines for gain‐of‐function analyses.

Seedlings of *GAD2ΔC* Line #97 had a yellowish phenotype until 4–5 d after germination, and then turned green and grew similar to wild type. As a result, we were unable to obtain stable T3 homozygous lines for further analyses because of gene silencing. *GAD2ΔC* Line #33 had the yellowish phenotype in the leaves from germination to 3 weeks in the T3 progenies. We compared the GAD2ΔC expression level and GABA content in the yellow leaves and green leaves of line *GAD2ΔC* #33 and found that the yellowish phenotype was tightly related to GAD2ΔC expression level and the GABA content (Figure 6D). The GAD2ΔC protein was much more abundant in the yellow leaves of *GAD2ΔC #*33 than in the green leaves. Likewise, the GABA content in the yellow leaves was about 7‐fold higher than in the green leaves of *GAD2ΔC* #33 and about 350‐fold higher than in wild‐type leaves (Figure 6E). This result indicated that the increased GABA level was tightly related to the yellowish phenotype and that high levels of GABA have a negative effect on vegetative growth.

**Figure 7:**
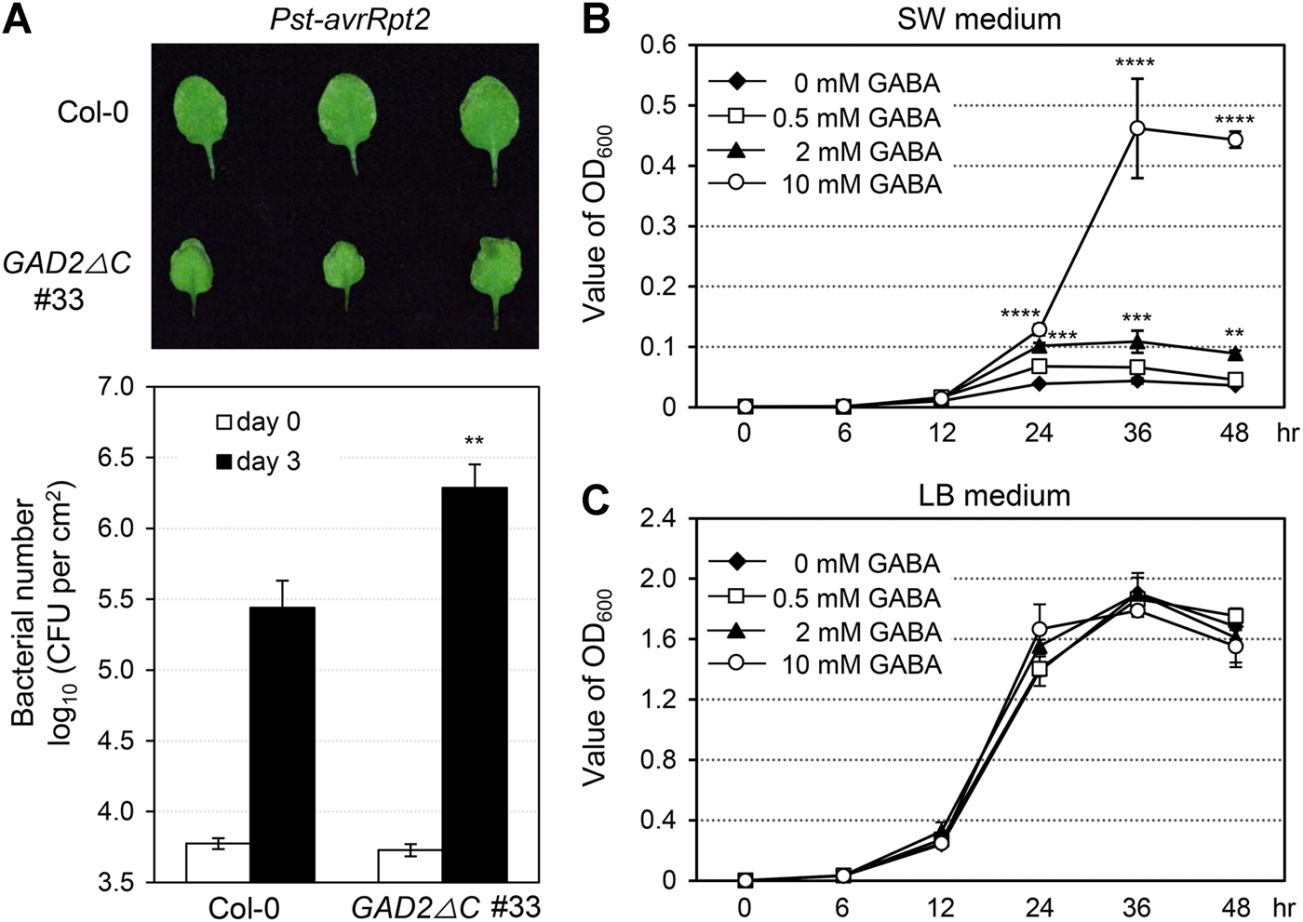
**Over‐accumulation of γ‐aminobutyric acid (GABA) is associated with decreased resistance to *Pst‐avrRpt2* in *glutamate decarboxylase (GAD)2ΔC* transgenic plants** (**A**) Three‐week‐old plant leaves were infiltrated with *Pst‐avrRpt2* (OD_600_ = 0.001). The bacteria were quantified 0 and 3 days post‐inoculation (dpi). Leaves used for bacterial counting in Col‐0 and *GAD2ΔC* #33 were from the same position. Student's *t*‐test was used to compare different genotypes with the same treatment at any certain time point (**P < 0.01). Error bars indicate *SD* (*n* = 3). CFU, colony‐forming units. (**B**–**C**) *Pst‐avrRpt2* (final concentration OD_600_ = 0.001) and different concentrations of GABA (0, 0.5, 2, or 10 mmol/L GABA) were added to plant culture medium (swimming medium, SW) (**B**) or Luria‐Bertani medium (**C**), respectively. Bacterial growth was measured at indicated time points and was analyzed by one‐way analysis of variance. Asterisks indicate statistical difference (**P < 0.01; ***P < 0.001; ****P < 0.0001). Error bars indicate *SD* (*n* = 3).

We also found that the elevated GABA levels and yellowish phenotype in the *GAD2ΔC* #33 plants were associated with a decrease in resistance to *Pst‐avrRpt2* (Figure 7A). Since *Cladosporium fulvum*, a fungal pathogen restricted to the intercellular space, can use GABA as a nitrogen source to support its growth (Solomon and Oliver 2001, 2002), we examined whether GABA has the potential to support bacterial pathogen growth as an N/C source. We examined *Pst‐avrRpt2* proliferation *in vitro* in the swimming medium (the liquid medium used for plant seedling culture in this study) supplied with GABA. *Pst‐avrRpt2* was inoculated at the initial population of OD_600_ = 0.001, equal to the concentration used for pathogen disease assay. The bacterial population reached an OD_600_ of 0.04 when no GABA was added in the medium (Figure 7B). Exogenous supplementation with 2 mmol/L GABA significantly promoted pathogen growth. When 10 mmol/L GABA was supplied to the swimming medium, the pathogen proliferated to an OD_600_ of 0.46 at 36 h (Figure 7B). This differential growth rate of *Pst‐avrRpt2* seen in the swimming medium supplied with different concentrations of GABA was not seen when GABA was added to Luria‐Bertani (LB) medium, which is already rich in N/C sources (Figure 7C). These results indicated that the bacterial pathogen is able to utilize GABA as an N/C source when nutrients are insufficient. Taken together, high levels of over‐accumulation of GABA could lead to the suppression of plant vegetative growth and the promotion of bacterial pathogen colonization.

## DISCUSSION

### The level of GABA is important to plant resistance

Interest in the role of GABA in plant immunity has recently increased because of its rapid induction in plants during their interaction with pathogens. However, the role of GABA in defense responses remains undefined because of the lack of loss‐of‐function genetic evidence. In this report, we found that the *gad1/2/4* triple and *gad1/2/4/5* quadruple mutants, in which the GABA level is extremely low, are more susceptible to both *Pst* and *Pst‐avrRpt2* infection (Figure 5C), indicating a positive role of GABA in plant immunity. Gamma‐aminobutyric acid may play roles for both the pathogen and the host. Gamma‐aminobutyric acid uptake by *Pst* can repress the expression of *hrp* genes, which encode components of the Type III Secretion System (T3SS), resulting in reduced bacterial virulence (Park et al. 2010; McCraw et al. 2016). In plants, GABA has both metabolic and signaling roles, which may both influence plant resistance. The GABA shunt contributes to both the TCA cycle and the respiratory electron transfer chain by generating succinate and nicotinamide adenine dinucleotide ‐ hydrogen through SSADH activity (Shelp et al. 1999). Although the levels of GABA in the *gad1/2/4* triple and *gad1/2/4/5* quadruple mutants are extremely low, the growth and development of these two mutants are indistinguishable from the wild type, indicating that the reduced GABA level has no significant influence on primary C/N metabolism under normal conditions. During *avrRpt2‐*ETI, GABA induction was associated with increased levels of several other amino acids, including alanine (Ala), glutamate (Glu), serine (Ser), and threonine (Thr). When GABA biosynthesis is blocked in the *gad1/2/4* triple and *gad1/2/4/5* quadruple mutants, the induction of these four amino acids was compromised. In particular, in the *gad1/2/4* and *gad1/2/4/5* mutants the level of Ala was only ~30% of that in wild type after *Pst‐avrRpt2* infection (Figure S4). This indicated that the GABA level is closely related to primary metabolism during *avrRpt2*‐ETI. We propose that the impaired primary metabolism could at least partially lead to the compromised resistance to pathogen infection of the *gad1/2/4* triple and *gad1/2/4/5* quadruple mutants.

The discoveries that GABA regulates the anion transporter TaALMT1 and that ALMT has a putative GABA‐binding site identified GABA as a possible signaling molecule in plants (Ramesh et al. 2015). *At*ALMT12 is a malate‐sensitive component of the R‐type anion channel in guard cells of *Arabidopsis*, and it is required for efficient stomatal closure (Meyer et al. 2010). Bacterial pathogen‐induced stomatal immunity is closely related to changes in malate content in the guard cells, and this response is regulated by the MPK3/6 cascade (Su et al. 2017). Further study is needed to determine if the reduced GABA levels in the *gad1/2/4* triple and *gad1/2/4/5* quadruple mutants might also impair stomatal closure during pathogen infection.

Although genetic evidence revealed that a reduced level of GABA compromised plant resistance to *Pst* and *Pst‐avrRpt2* (Figure 5C), *GAD2ΔC* transgenic plants were more susceptible to *Pst‐avrRpt2*, in which GABA accumulates to a high level (Figure 7A). However, the dramatic increase in GABA levels results in the arrest of plant growth in the transgenic plants (Figure 6C). This is consistent with the observations that overexpression of *GAD* resulted in a dwarf phenotype in *Arabidopsis*, tobacco, and tomato (Michaeli and Fromm 2015). As a result, it is difficult to reach a definite conclusion about the role of increased GABA in the immune response at this stage. Nevertheless, this result further highlights the importance of balanced levels of GABA in a plant. This is further complicated by how the pathogen responds to GABA: high levels of GABA do not negatively affect bacteria proliferation *in vitro* (Figure 7C), and GABA can serve as a N/C resource to support *Pst* growth (Figure 7B). In the future, more detailed work is needed to dissect the relationship between the magnitude of GABA content and the plant resistance response from both sides of the interaction between host and pathogen.

### **Contribution of each** *GAD* **gene to GABA biosynthesis and their differential regulation in plant immunity**

In this report, we found that *GAD1*, *GAD2*, and *GAD4* function together to increase GABA levels during plant defense responses. It has been reported that *GAD1* is abundantly expressed in root, *GAD2* is constitutively expressed in all organs, while the basal expression of *GAD4* is pretty low (Bouche et al. 2004, Miyashita and Good 2008). Our data from the GUS reporter lines confirmed this (Figure S1). Genetic evidence indicated that *GAD2* is the major *GAD* gene responsible for both basal and pathogen‐induced GABA levels in shoots, while *GAD1* and *GAD4* also contribute significantly (Figure 5A). The expression of these three *GAD* genes is differentially regulated. In response to *Pst‐avrRpt2* infection, expression of *GAD1* and *GAD4* is induced, while expression of *GAD2* is downregulated (Figure 3). Differential regulation of different *GAD* genes also exists in other processes. During fruit development and ripening, the transcript level of *GAD2* is enhanced in parallel with genes of central metabolism (Fait et al. 2008). In response to hypoxia, expression of *GAD2* is suppressed, expression of *GAD4* is induced, and there is no change in *GAD1* expression (Miyashita and Good 2008). All of these results suggest that the levels of GABA are tightly controlled through the modulation of transcript abundance of the different *GAD* genes during different growth stages or under different stress conditions.

Both *Pst‐avrRpt2* infection and activation of MPK3/MPK6 suppress *GAD2* expression and induce the expression of *GAD1/GAD4*. However, the induction/suppression kinetics are different. Continuous activation of MPK3/MPK6 in *DD* transgenic plants resulted in a ~98% reduction in *GAD2* transcript abundance and a ~23,000‐fold induction in *GAD4* transcripts (Figure 1), indicating that the most abundant GAD enzymes were translated from the induced *GAD4* transcripts. It seems likely that the increase in *GAD4* transcripts would exceed the decrease in *GAD2* transcripts after MPK3/MPK6 activation. In response to *Pst‐avrRpt2* infection, there was only a ~42% reduction in *GAD2* transcript levels, and only a ~100‐fold induction (from the very low basal level) in *GAD4* transcripts (Figure 3). With these changes in transcript levels, the increase in *GAD4* transcripts may not compensate for the loss of *GAD2* transcripts, resulting in *GAD2* messenger RNAs remaining the most abundant *GAD* transcripts in infected leaves. The differential induction/suppression kinetics of the *GAD* genes may explain why *Pst‐avrRpt2*‐induced GABA is mainly dependent on *GAD2/1/4*, while in DEX‐treated *DD* plants, induction of GABA is mainly dependent on *GAD1/4*.

Gamma‐aminobutyric acid biosynthesis was induced to a very high level after long‐lasting activation of the MPK3/MPK6 signaling cascade (Figure 1D). During *avrRpt2*‐ETI, loss of function of MKK4/MKK5‐MPK3/MPK6 or WRKY33 resulted in dramatically decreased induction of the *GAD1* and *GAD4* genes (Figure 4A, B), suggesting important transcriptional regulation of these two GAD genes by this MAPK cascade during ETI. However, the GABA concentration in *mpk3*, *mpk6*, and *mkk4/mkk5* mutants after *Pst‐avrRpt2* infection was not reduced, and was even higher in the *mkk4/mkk5* mutant in comparison to the wild type (Figure S6). We found that the expression of *GAD2* is not suppressed in these mutants when inoculated with pathogen as it is in the wild type (Figure S3), which might be one reason for the slightly higher GABA level in these mutants after *Pst‐avrRpt2* infection. Alternatively, there might be other pathway(s) that regulate GABA biosynthesis during this process. Most GAD proteins in plants contain a Ca^2+^/CaM‐binding domain, implying their potential regulation by a Ca^2+^/CaM signaling pathway at the protein level (Baum et al. 1993; Chen et al. 1994; Baum et al. 1996), a regulatory step which needs to be studied further.

## MATERIALS AND METHODS

### Plant materials and growth conditions

Mutant and wild‐type plants in *Arabidopsis thaliana* Columbia (Col‐0) ecotype were used in all experiments. T‐DNA insertion alleles and transgenic lines of *mpk3* (Salk_151594), *mpk6‐3* (Salk_127507), *wrky33‐2* (GABI_324B11), *DD (GVG‐NtMEK2* ^*DD*^ *)*, *DD mpk3*, *DD mpk6*, *rps2 (rps2‐101C)* were previously described (Liu and Zhang 2004; Wang et al. 2007; Guan et al. 2015). T‐DNA insertion mutant alleles of *gad1‐1* (SALK_017810C), *gad2* (GABI_474E05), *gad4* (SALK_106240C) and *gad5* (SALK_203883C) were ordered from the ABRC.

Swimming plants were grown in 20 mL gas chromatography vials with 6 mL of half‐strength Murashige and Skoog (MS) liquid medium in a growth chamber under continuous light (70 μE/m^2^/s) as described (Ren et al. 2008). Twelve‐d‐old seedlings were used for experiments. Seeds were imbibed at 4°C for 3 d and then grown in the soil at 22°C in a growth chamber with a 14 h light cycle (100 μE/m^2^/s) and 80% relative humidity (Guan et al. 2015).

### Gamma‐aminobutyric acid extraction and quantification

Plant tissues were harvested, frozen in liquid nitrogen, and stored at ‐80°C. Samples (30 to 50 mg) were ground into powder, homogenized in 4% salicylic acid (0.7 mL) by a pestle, then ultrasonicated for 20 s at 100 watts. The acid‐soluble fraction was separated by centrifugation (4°C, 10,000 × *g*) for 10 min. Gamma‐aminobutyric acid concentrations were measured in the supernatant after filtration (0.22 μm). Gamma‐aminobutyric acid and other free amino acids were analyzed using a Hitachi Automatic Amino Acid Analyzer (L‐8900). The amino acids were detected at 570 nm. Quantification was carried out on the basis of the chromatogram peaks (Le Boucher et al. 1997).

### Quantitative real‐time polymerase chain reaction analysis

Plant RNA was extracted using the TRIzol reagent (Invitrogen). After treatment with DNase (Invitrogen), 1 μg total RNA was used to synthesize first‐strand complementary DNA. Real‐time quantitative polymerase chain reaction (PCR) analyses were conducted by a real‐time PCR machine (Eppendorf) as described (Ren et al. 2008). Gene expression levels were calculated as percentages referring to the EF1α transcript. The primers that were used in real‐time PCR are presented in Table S1.

### Generation of mutant lines and transgenic plants

Single *gad* mutants were crossed to obtain double, triple and quadruple mutants. The specific primers for different genes used for mutant genotyping and confirmation are listed in Table S3. To generate GUS reporter lines for the five *GAD* genes, the GAD promoters were amplified by nested PCR. The first and second PCR primer pairs used for each gene are shown in Table S2. The PCR fragment of each gene was cloned into the pBIB binary vector to generate pBIB*‐P*_*GAD*_ *:GUS* constructs. To generate 35S promoter‐driven truncated *GAD* (*35s:Flag‐GAD1ΔC/GAD2ΔC/GAD4ΔC*) in *Arabidopsis*, the *GAD* CDS was amplified by nested PCR. The first and second PCR primer pairs used for amplifying each gene are shown in Table S2. The PCR fragment was cloned into the pBlueScript II KS vector to generate pBS‐*Flag‐GADs* constructs. Then deletion PCR was performed on pBS‐*Flag‐GADs* to generate the truncated forms of GADs. The DNA was end‐phosphorylated and ligated to generate pBS‐*Flag‐GADΔC*. The *Xho*I‐ and *Spe*I‐digested *Flag‐GADΔC* were then ligated to the pBId binary vector to generate pBId*‐35S:Flag‐GADΔC* constructs. All binary vectors were transformed into *Agrobacterium* strain GV3101. *Arabidopsis* transformation was by the floral dip procedure (Clough and Bent 1998). Transgenic plants were selected by kanamycin resistance on agar plates.

### Protein extraction and western blot analysis

Protein was extracted from shoots or leaves of Arabidopsis and stored at ‐80°C as described (Liu and Zhang 2004). The concentration of protein extracts was determined using the Bio‐Rad protein assay kit with bovine serum albumin as the standard. Transgenic proteins were analyzed by immunoblot using anti‐Flag antibody.

### **Pathogen disease assay and** *in vitro* **growth assay**

For pathogen disease assay, ~3‐week‐old Col‐0 and *gad* mutant plants grown under a short‐day cycle (10 h light/14 h dark) were used. The fifth and sixth leaves were infiltrated with *Pst‐avrRpt2* (OD_600_ = 0.001) in 10 mmol/L MgCl_2_. Leaves were detached and washed with 0.02% Silwet L‐77 before leaf discs were punched out for bacterial growth assays as previously described (Guan et al. 2015; Su et al. 2017). For *in vitro* assays, bacteria were scraped off the plates and suspended in liquid one‐half‐strength MS or LB medium to OD_600_ = 0.1. *Pst‐avrRpt2* (10 μL) was added to a tube with 1 mL of either media to a final OD_600_ = 0.001. The *Pst* suspension was cultured with shaking at 28 °C. The OD_600_ was measured at the indicated times to track growth.

### β‐glucuronidase histochemical analysis

Samples were harvested and incubated in GUS staining buffer for 2 h (for seedlings) or 12 h (for flowers) at 37 °C. The samples were then fixed in FAA (5% ethanol, 5% acetic acid, and 3.7% formaldehyde) for 1 h, cleared in 20% lactic acid and 20% glycerol, and observed on a Nikon Eclipse 80i microscope as described (Wang et al. 2007).

### Statistical analyses

At least two independent repetitions were performed for experiments with multiple time points. For single time point experiments, at least three independent repetitions were done. Results from one of the independent repeats that gave similar results are shown. Statistical analysis was performed using GraphPad Prism 8.0 (http://www.graphpad.com/). Student's *t*‐test was used to determine whether the difference between two groups of data was statistically significant at certain time points. Asterisks above the columns indicate statistical significance. When more than two samples are compared, multiple comparisons post one‐way analysis of variance (ANOVA) was performed. Two‐way ANOVA analysis with multiple comparisons was carried out when time‐course data of different treatments/genotypes were compared. Different letters above the data points are used to indicate differences that are statistically significant.

### Accession numbers

Sequence data from this article can be found in the GenBank/EMBL data libraries under the following accession numbers: At3g45640 (*MPK3*), At2g43790 (*MPK6*), At1g51660 (*MKK4*), and At3g21220 (*MKK5*), At2g38470 (*WRKY33*), At5g17330 (*GAD1*), At1g65960 (*GAD2*), At2g02000 (*GAD3*), At2g02010 (*GAD4*), and At3g17760 (*GAD5*).

## AUTHOR CONTRIBUTIONS

X.D., J.X., and S.Z. designed the project. X.D. performed most of the experiments with the help of X.X., Y. L., L.Y., and Y. Z. X.D., S.Z., and J.X. analyzed the results and wrote the manuscript. All authors read and approved the manuscript.

## Supporting information

Additional Supporting Information may be found online in the supporting information tab for this article: http://onlinelibrary.wiley.com/doi/10.1111/jipb.12974/suppinfo

**Figure S1**. Expression patterns of *GAD* promoters at different developmental stages in *Arabidopsis*
Transgenic _*Pro*_ *GADs:GUS* reporter lines were stained at the indicated time after germination. dpg, d post‐germination. (**A**) Bar = 250 μm. (**B–F**) Bar = 0.5 cm.
**Figure S2**. Identification of *gad* mutants
(**A–D**) Schematic diagrams of T‐DNA insertion sites of each *gad* mutant. (**E**) Transcription level confirmation of *GAD* presence by reverse transcription polymerase chain reaction in wild‐type Col‐0, the *gad* quadruple mutant, and the *DD* mutant with dexamethasone (DEX) treatment. The sampled tissues were seedling shoot, flower and whole seedling.
**Figure S3**. Expression of *GAD2* in *mpk3*, *mpk6*, *mkk4 mkk5*, and *wrky3*3 mutants after *Pst‐avrRpt2* infection
Fourteen‐d‐old seedlings were collected at the indicated times after spray inoculation with *Pst‐avrRpt2* (OD_600_ = 0.4). Gene expression was quantified by real‐time polymerase chain reaction. Error bars indicate *SD* (*n* = 3).
**Figure S4**. Cellular levels of selected free amino acids in Col‐0, *gad1/2/4*, and *gad1/2/4/5* mutants after *Pst‐avrRpt2* inoculation
Fourteen‐d‐old seedlings of Col‐0, *gad1/2/4* and *gad1/2/4/5* mutants were sprayed with *Pst‐avrRpt2* (OD_600_ = 0.4). Samples were collected 18 h after treatment. Free amino acids were determined using the Amino Acid Analyzer. One‐way analysis of variance was performed to compare different genotypes with the wild type. Asterisks above the columns indicate statistical difference (**P  < 0.01; ***P  < 0.001; ****P  < 0.0001). Error bars indicate *SD* (*n* = 3). FW, fresh weight.
**Figure S5**. Schematic representation of the γ aminobutyric acid (GABA) shunt in *Arabidopsis*
GAD, glutamate decarboxylase; GABP, GABA permease; GABA‐T/POP2, γ‐aminobutyric acid transaminase; SSADH, succinic semialdehyde dehydrogenase; GDH, glutamate dehydrogenase. Alanine is a by‐product of the GABA shunt.
**Figure S6**. Gamma‐aminobutyric acid (GABA) concentrations in Col‐0, *mpk3*, and *mpk6* single mutants and *mkk4 mkk5* double mutant after *Pst‐avrRpt2* spray
Shoots of 14‐d‐old, soil‐grown seedlings were collected at indicated times after spraying with *Pst‐AvrRpt2* (OD_600_ = 0.4). One‐way analysis of variance (**A**) was applied when three genotypes were compared, and Student's *t*‐test (**B**) was performed when two genotypes were compared at certain time points (*P < 0.05), Error bars indicate *SD* (*n* = 3). FW, fresh weight.
**Table S1**. Primer pairs for quantitative polymerase chain reaction
**Table S2**. Primer pairs used for cloning
**Table S3**. Primer pairs used for mutant genotyping and cDNA confirmation
