# Supplementary material for: Induction of γ‐aminobutyric acid plays a positive role to Arabidopsis resistance against Pseudomonas syringae
Source: J Integr Plant Biol. 2020 Jun 26;62(11):1797–812. doi: 10.1111/jipb.12974 (PMC7689811; doi:10.1111/jipb.12974)
Supplement: Supplementary file 1 — Figure S1. Expression patterns of GAD promoters at different developmental stages in Arabidopsis Transgenic Pro GADs:GUS reporter lines were stained at the indicated time after germination. dpg, d post‐germination. (A) Bar = 250 μm. (B–F) Bar = 0.5 cm. Figure S2. Identification of gad mutants (A–D) Schematic diagrams of T‐DNA insertion sites of each gad mutant. (E) Transcription level confirmation of GAD presence by reverse transcription polymerase chain reaction in wild‐type Col‐0, the gad quadruple mutant, and the DD mutant with dexamethasone (DEX) treatment. The sampled tissues were seedling shoot, flower and whole seedling. Figure S3. Expression of GAD2 in mpk3, mpk6, mkk4 mkk5, and wrky33 mutants after Pst‐avrRpt2 infection Fourteen‐d‐old seedlings were collected at the indicated times after spray inoculation with Pst‐avrRpt2 (OD600 = 0.4). Gene expression was quantified by real‐time polymerase chain reaction. Error bars indicate SD (n = 3). Figure S4. Cellular levels of selected free amino acids in Col‐0, gad1/2/4, and gad1/2/4/5 mutants after Pst‐avrRpt2 inoculation Fourteen‐d‐old seedlings of Col‐0, gad1/2/4 and gad1/2/4/5 mutants were sprayed with Pst‐avrRpt2 (OD600 = 0.4). Samples were collected 18 h after treatment. Free amino acids were determined using the Amino Acid Analyzer. One‐way analysis of variance was performed to compare different genotypes with the wild type. Asterisks above the columns indicate statistical difference (**P < 0.01; ***P < 0.001; ****P < 0.0001). Error bars indicate SD (n = 3). FW, fresh weight. Figure S5. Schematic representation of the γ aminobutyric acid (GABA) shunt in Arabidopsis GAD, glutamate decarboxylase; GABP, GABA permease; GABA‐T/POP2, γ‐aminobutyric acid transaminase; SSADH, succinic semialdehyde dehydrogenase; GDH, glutamate dehydrogenase. Alanine is a by‐product of the GABA shunt. Figure S6. Gamma‐aminobutyric acid (GABA) concentrations in Col‐0, mpk3, and mpk6 single mutants and mkk4 mkk5 double mutant after Pst‐ [file JIPB-62-1797-s001.docx]

**Supplemental Information**


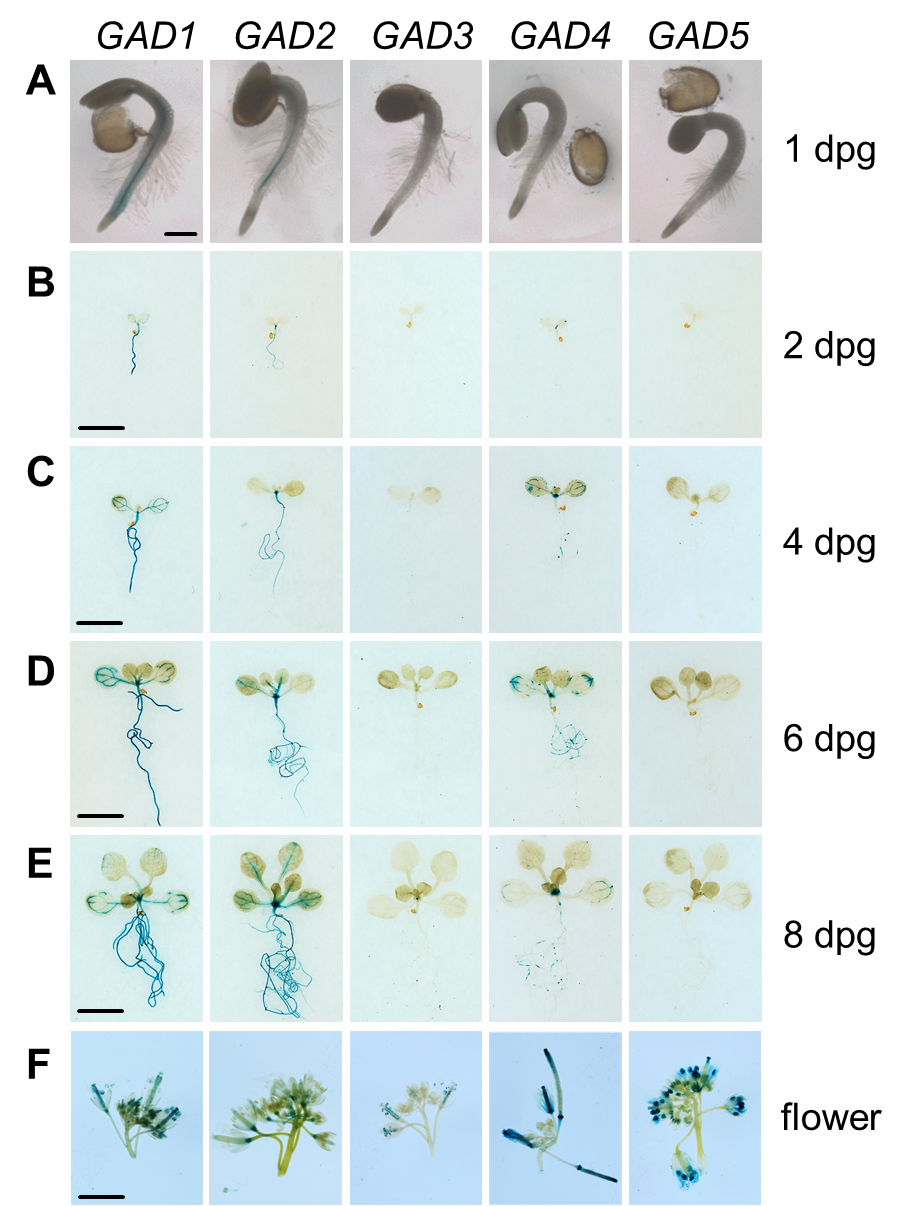


**Figure S1.** Expression patterns of *GAD* promoters at different developmental stages in *Arabidopsis*

Transgenic *_Pro_GADs:GUS* reporter lines were stained at the indicated time after germination. dpg, day post germination. (A) Bar = 250 μm. (B-F) Bar = 0.5 cm.


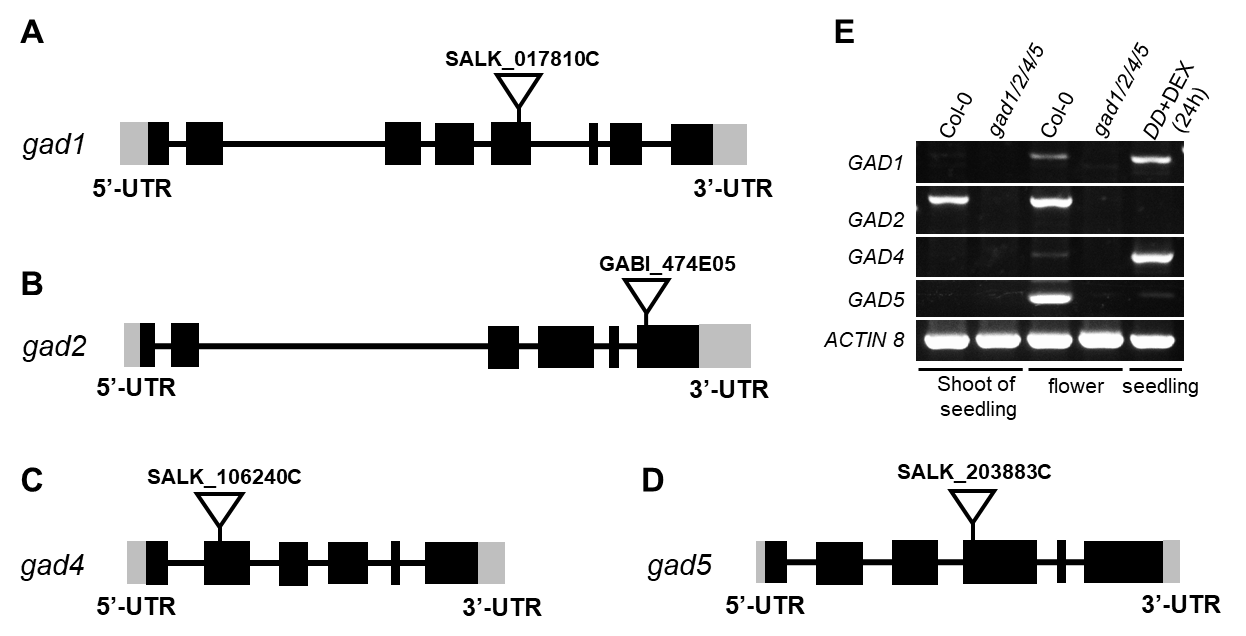


**Figure S2.** Identification of *gad* mutants

(A-D) Schematic diagrams of T-DNA insertion sites of each *gad* mutant. (E) Transcription level confirmation of *GAD* presence by reverse transcription polymerase chain reaction in wild-type Col-0, the *gad* quadruple mutant, and the *DD* mutant with DEX treatment. The sampled tissues were seedling shoot, flower and whole seedling.


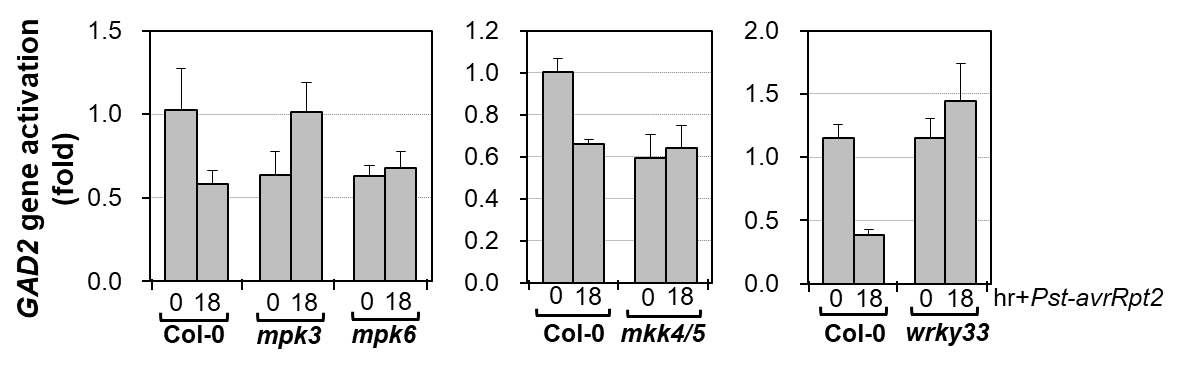


**Figure S3.** Expression of *GAD2* in *mpk3*, *mpk6*, *mkk4 mkk5*, and *wrky3*3 mutants after *Pst-avrRpt2* infection

Fourteen-day-old seedlings were collected at the indicated times after spray-inoculation with *Pst-avrRpt2* (OD_600_ = 0.4). Gene expression was quantified by RT-qPCR. Error bars indicate *SD* (*n* = 3).

**
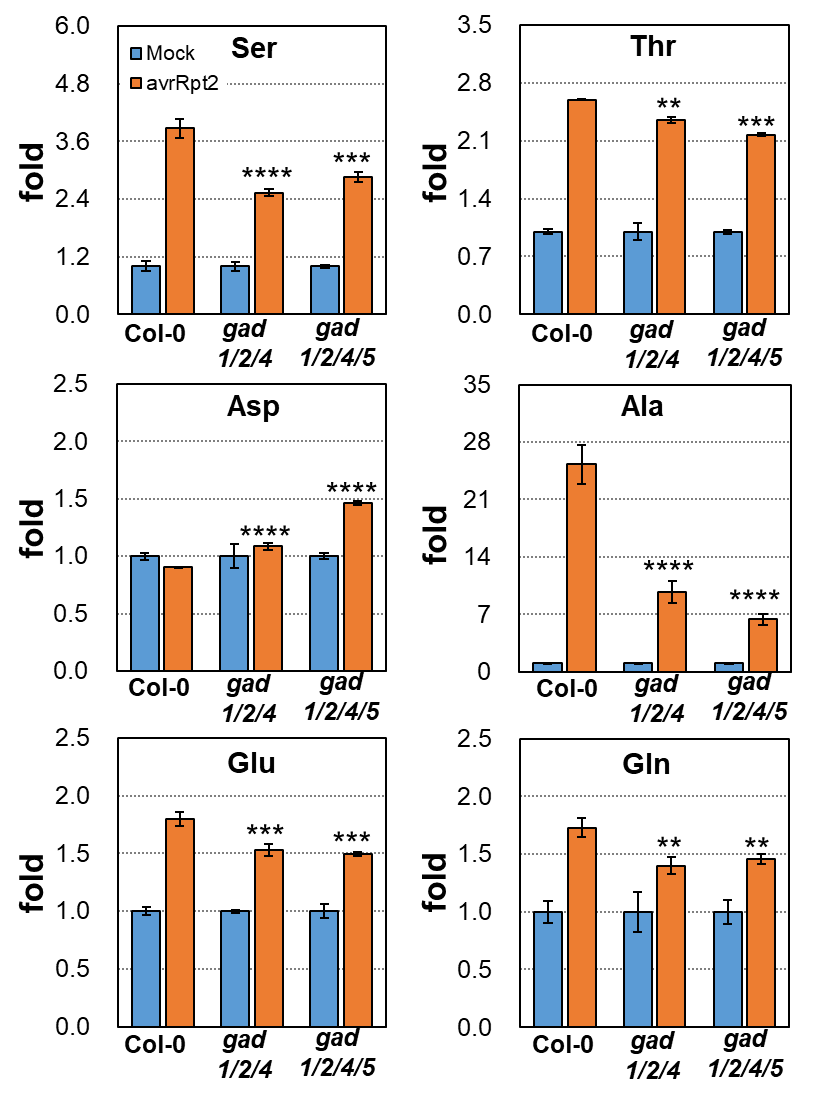
**

**Figure S4.** Cellular levels of selected free amino acids in Col-0, *gad1/2/4*, and *gad1/2/4/5* mutants after *Pst-avrRpt2* inoculation

Fourteen-day-old seedlings of Col-0, *gad1/2/4* and *gad1/2/4/5* mutants were sprayed with *Pst-AvrRpt2* (OD_600_ = 0.4). Samples were collected 18 h after treatment. Free amino acids were determined using the Amino Acid Analyzer. One-way ANOVA was performed to compare different genotypes with the wild type. Asterisks above the columns indicate statistical difference (**, *P* <0.01; ***, *P* <0.001; ****, *P* <0.0001). Error bars indicate *SD* (*n* = 3). FW, fresh weight.


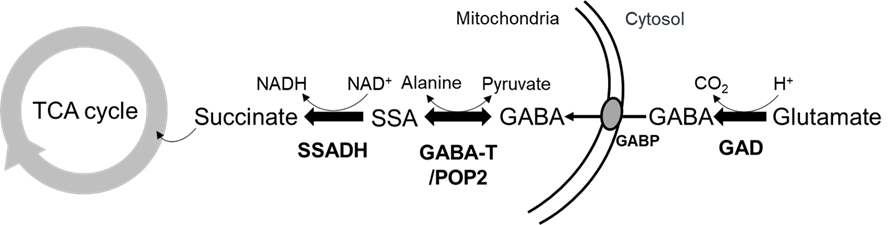


**Figure S5.** Schematic representation of the GABA shunt in *Arabidopsis*

GAD, glutamate decarboxylase; GABP, GABA permease; GABA-T/POP2, g-aminobutyric acid transaminase; SSADH, succinic semialdehyde dehydrogenase; GDH, glutamate dehydrogenase. Alanine is a by-product of the GABA shunt.


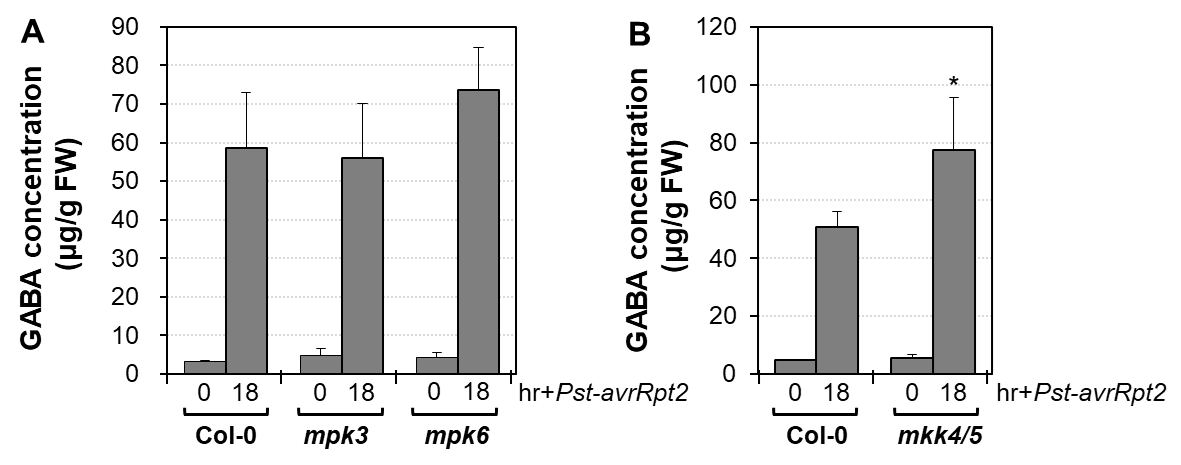


**Figure S6.** GABA concentrations in Col-0, *mpk3*, and *mpk6* single mutants and *mkk4 mkk5* double mutant after *Pst-avrRpt2* spray

Shoots of 14-day-old, soil-grown seedlings were collected at indicated times after spraying with *Pst-AvrRpt2* (OD_600_ = 0.4). One-way ANOVA (A) was applied when three genotypes were compared, and student’s *t*-test (B) was performed when two genotypes were compared at certain time points (*, *P* <0.05), Error bars indicate *SD* (*n* = 3). FW, fresh weight.

| **Table S1.** Primer pairs used for q-PCR | |
| --- | --- |
| **Name** | **Sequence** |
| *GAD1* qPCR F | ATGAGCTTCCTTCGAGAGTGAT |
| *GAD1* qPCR B | ACTTCTTCCAGCCAGTGATGAT |
| *GAD2* qPCR F | TGTGTTCTTCTTCCTCCTCAGA |
| *GAD2* qPCR B | TGTAGTGCGAACATAGCGAGAT |
| *GAD3* qPCR F | TGCACATTTTTCCCTTTACTTTT |
| *GAD3* qPCR B | TCGAATCGTGAGATAGAGTTGC |
| *GAD4* qPCR F | TCTTGTTCATTTCAAACCCAAA |
| *GAD4* qPCR B | TTTCGAATCGTGGAAGAGAGTT |
| *GAD5* qPCR F | AATGGTACTCGCAACCAACTCT |
| *GAD5* qPCR B | CATTGATCACTTGATAAGCAGCA |
| *EF1α* F | GATTGCCACACCTCTCACATT |
| *EF1α* B | CATACCAGCGTCACCATTCTT |

| **Table S2.** Primer pairs used for cloning | | | |
| --- | --- | --- | --- |
| **Primer name** | **Application** | **Sequence** | |
| GAD1 pro F1 | 1st PCR for cloning | | GTTGGATGTTGAAGAGGAGAATC |
| GAD1 pro B1 | 1st PCR for cloning | | TGAAGTACGGACGTAACGTGAT |
| GAD1 pro *Sal* I F2 | 2nd PCR for cloning | | ACGCGTCGACGTTGGATGTTGAAGAGGAGAATC |
| GAD1 pro B2 | 2nd PCR for cloning | | CACGGAGATGAGAGAGAGAGAA |
| GAD2 pro F1 | 1st PCR for cloning | | CAATCCAGGGTATTGCACCTAT |
| GAD2 pro B1 | 1st PCR for cloning | | TTTGTCACACTCTGGTTCCATC |
| GAD2 pro *Hind* III F2 | 2nd PCR for cloning | | CCCAAGCTTCAATCCAGGGTATTGCACCTAT |
| GAD2 pro B2 | 2nd PCR for cloning | | CTTTGTTTCTGTTTAGTGAAAGAGA |
| GAD3 pro F1 | 1st PCR for cloning | | GTGAACGGTTTGATTTGAACCT |
| GAD3 pro B1 | 1st PCR for cloning | | ACATAACGGGAAGCAAAAGTTG |
| GAD3 pro *Hind* III F2 | 2nd PCR for cloning | | CCCAAGCTTGTGAACGGTTTGATTTGAACCT |
| GAD3 pro B2 | 2nd PCR for cloning | | CTTCTTGAAATGAGCTAAAGAAAAG |
| GAD4 pro F1 | 1st PCR for cloning | | CCATAGCACACTTTTCCCCTAA |
| GAD4 pro B1 | 1st PCR for cloning | | GGACGTAACGAGAAGCAAAAGT |
| GAD4 pro *Hind* III F2 | 2nd PCR for cloning | | CCCAAGCTTCCATAGCACACTTTTCCCCTAA |
| GAD4 pro B2 | 2nd PCR for cloning | | GTCTTCTTTCAACTTCGAACTTTGAT |
| GAD5 pro F1 | 1st PCR for cloning | | GGCACAAGCGGAATAATGTAAT |
| GAD5 pro B1 | 1st PCR for cloning | | AAAATGAGGCAAAACTCTCTGG |
| GAD5 pro *Sal* I F2 | 2nd PCR for cloning | | ACGCGTCGACGGCACAAGCGGAATAATGTAAT |
| GAD5 pro B2 | 2nd PCR for cloning | | TGTGTTTGAGTTATGCTGAATTGTTG |
| GAD1 clo F1 | 1st PCR for cloning | | GTCTCTCATCATCCTCCATTCC |
| GAD1 clo B1 | 1st PCR for cloning | | CGATTAGCAGATACCACTCGTCT |
| GAD1 clo *Nde* I F2 | 2nd PCR for cloning | | CATATGGTGCTCTCCCACGCCGTAT |
| GAD1 clo B2 | 2nd PCR for cloning | | SAME AS GAD1 CLO B1 |
| GAD2 clo F1 | 1st PCR for cloning | | CATTGGACTAAGGAGCTTCCAC |
| GAD2 clo B1 | 1st PCR for cloning | | CACAAAGGCAACACACTTGCTTA |
| GAD2 clo *Nde* I F2 | 2nd PCR for cloning | | CATATGGTTTTGACAAAAACCGCAAC |
| GAD2 clo *Sal* I B2 | 2nd PCR for cloning | | GTCGACTTAGCACACACCATTCATCTTC |
| GAD4 clo F1 | 1st PCR for cloning | | ATGGTTTTGTCTAAGACAGTTTCC |
| GAD4 clo B1 | 1st PCR for cloning | | GCAAATTGTGTTCTTGTTGGTCT |
| GAD4 clo *Nde* I F2 | 2nd PCR for cloning | | CATATGGTTTTGTCTAAGACAGTTTCC |
| GAD4 clo B2 | 2nd PCR for cloning | | TTAGCAAATTGTGTTCTTGTTGGTCT |
| GAD1 C-del F | deletion PCR | | TAAGGGATCCACTAGTTCTAGAG |
| GAD1 C-del B | deletion PCR | | CTTCTTCACCGTGACCATCAA |
| GAD2 C-del F | deletion PCR | | TAAGTCGACGGGCTGCAGGAA |
| GAD2 C-del B | deletion PCR | CTCCTTTACATTTTCCGCGATC | |
| GAD4 C-del F | deletion PCR | TAAGGGGGATCCACTAGTTC | |
| GAD4 C-del B | deletion PCR | CGTCTTCTTAACACCGTTAAC | |

| **Table S3.** Primer pairs used for mutant genotyping and cDNA confirmation | | |
| --- | --- | --- |
| Primer name | Application | Sequence |
| GAD1 mut F | genotyping | GGAGCCAATGTTCAAGTAACG |
| GAD1 mut B | genotyping | GTGGACTGACTTACCTCGTGG |
| GAD2 mut F | genotyping | GATCAACCCACTTTCACTCTCA |
| GAD2 mut B | genotyping | GTGTTTGACTTGCTTGGCATAA |
| GAD4 mut F | genotyping | CAATAAAAAGATGACGGTCGG |
| GAD4 mut B | genotyping | TTGAACCGGAAATTGAGTCAC |
| GAD5 mut F | genotyping | CTGCTATTGGGTGTGGAACTG |
| GAD5 mut B | genotyping | TCCTTCTCTTAGCCTCCTTGC |
| JL202 | genotyping | CATTTTATAATAACGCTGCGGACAT |
| LB-sail | genotyping | TTCATAACCAATCTCGATACAC |
| LBb1.3 | genotyping | ATTTTGCCGATTTCGGAAC |
| DD LP | genotyping | CCCTCTGATGTGTTGTTGTTC |
| DD RP | genotyping | GTTAAGTCGCCCAATGTTGTC |
| DD border | genotyping | CAAACTGGAACAACACTCAACC |
| GAD1 cDNA F | expression | CACATCTATTCAATGCACCGTTA |
| GAD1 cDNA B | expression | ACTTCTTCCAGCCAGTGATGAT |
| GAD2 cDNA F | expression | TCGCTATGTTCGCACTACACTT |
| GAD2 cDNA B | expression | TTCCTCTCCTTCACAAACTTCC |
| GAD4 cDNA F | expression | SAME AS GAD4 CLO F1 |
| GAD4 cDNA B | expression | SAME AS GAD4 CLO B1 |
| GAD5 cDNA F | expression | AATGGTACTCGCAACCAACTCT |
| GAD5 cDNA B | expression | TTACCGCCAAATGGTCTAAATC |
| Actin8 FP | expression | GAGCACCCGGTTCTACTTACC |
| Actin8 RP | expression | GGGAAGCAAGGATAGAACCAC |
